# Case study: improving the management of eye care programmes

**Published:** 2008-12

**Authors:** Kolawole Olumide Ogundimu

**Affiliations:** Ophthalmologist and Head of Programme Development, West Africa – East Region, Sightsavers International, Accra, Ghana.

**Figure:**
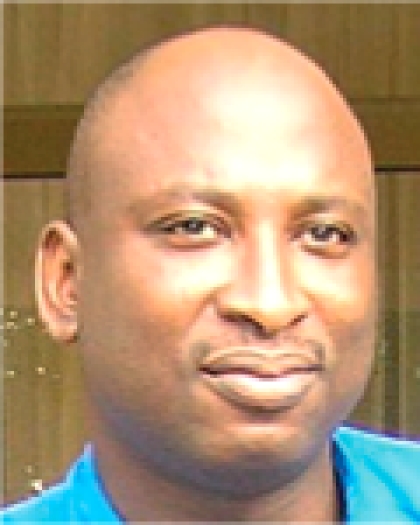


Nigeria is a federation of 36 administrative States, which each have a mandate to deliver health care to their population. Sightsavers International has been supporting four State ministries of health (Cross River, Kaduna, Kwara, and Sokoto) in implementing programmes designed to provide comprehensive eye care services.

Previously, eye care had been limited to services in big hospitals in the largest cities, which were inaccessible to the general population.

These new eye care programmes have been very successful. The Kwara eye care programme (KWECP), for example, has increased the cataract surgical rate from 196 cataract operations per million per year in 2003 to 932 in 2007.

This article describes what we have learnt from our experience in managing these programmes and hopefully will provide you with suggestions to improve management.

## Participatory planning

Planning for the Nigeria eye care programmes was participatory, and VISION 2020 provided us with a clear vision and strategy.^1^ Three initial consultations with the State ministries of health were followed by meetings with the different stakeholders. These involved all relevant players in eye care at field level, all levels of government in health care delivery in the geographic region, and representatives of all health professionals involved in eye care.

Stakeholder meetings have been invaluable and helped to bring together what would otherwise have remained very diverse partners.

## Managing projects within a programme

An eye care programme often consists of various projects, designed to control different diseases within a finite period. This is due to disease focus, development priorities, and/or funding and management considerations.

The KWECP was running a Mectizan® distribution project, a vitamin A distribution project, and an eye care project under separate funding and management. Our experience showed that the people working with these various projects had a tendency to drift apart and work separately, that the different projects often resulted in conflicting pressures on the same staff, which resulted in inadvertent duplication of efforts and limited synergy.

**Figure:**
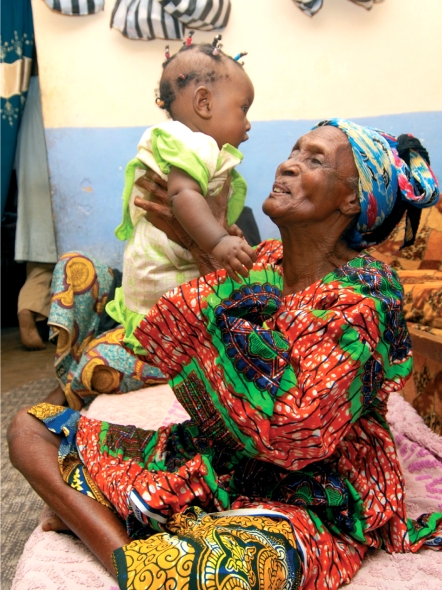
An older woman sees her granddaughter for the first time after cataract sugery. NIGERIA

To remedy this situation, we organised joint planning sessions between the management teams of the various projects under a unified State eye care programme, and we made sure that project reports were circulated to all other projects. Where possible, we tried to merge projects under one management team. We learnt that:

- Systems should be in place to ensure that different projects within the programme are complementary and synergistic.
- A project, which is for a finite period, is only one step in what will be a long-term programme. Elements of sustainability and long-term programme planning should be incorporated from the beginning.

## Managing human resources

Human resources are the most valuable asset of any eye care programme. Team building, continuous training, and motivation are essential aspects of human resource management^2^ (see Box on the right).

### Assessing human resources

In order to effectively manage and develop human resources, it is essential to start by:

- estimating the available pool of people who may be trained
- assessing the levels of competence of existing personnel, as they may need some extra training.

In some States, a dearth of qualified manpower to train has limited the development of human resources. For this reason, the development of middle and lower cadres, especially ophthalmic nurses, has been less successful than that of ophthalmologists.

We also found that most existing staff required re-training before they could effectively perform their roles in service delivery.

### The importance of teamwork

Moving from an individual approach to a team approach has been a key factor in improving output in the KWECR

The programme set up service teams responsible for eye health in defined geographic zones in the State. These teams comprised the various professionals required to deliver service; we used available staff and also organised training to fill identified gaps in human resources. We developed an approach centred on base hospitals and outreach clinics and surgery, and we instituted management committees and coordinating systems. We therefore moved away from the service delivery system that had previously been the norm, which was centred on a single hospital and ophthalmologist.

### Training

It is important to invest in quality training, not forgetting to add principles of management for those in leadership positions. Training should be targeted at building appropriate teams with the correct mix of professionals and support staff.^3^

We have had much success in the training of ophthalmologists and we set up a practical training centre for ophthalmologists in KWECP. Two factors were key to this success. Firstly, there existed a pool of general practitioners in the State services which were available for training — this is not always the case in sub-Saharan Africa and cannot be taken for granted. Secondly, we benefited from the presence of a motivated consultant ophthalmologist in the programme, who became the principal trainer and acted as a focal person and driver for the training programme, as well as for service delivery.

### Motivating staff

The team leader plays a vital role in keeping his/her staff motivated. Motivation involves the development and maintenance of a shared vision, appropriate training and acquisition of skills, the recognition of individual contribution, and a careful use of incentives.

Performance-linked financial incentives alone are usually not very good motivators and may lead to service or project failure if the incentives dry up.

In our programmes, motivation was essentially the result of:

- improving staff morale through a culture of support and encouragement
- providing quality equipment and adequate consumables
- ensuring a conducive environment for service delivery
- providing the training necessary for the tasks at hand
- the incentive, with good performance, of further training and development of skills.

## Managing infrastructure and technology

Before planning a project, it is important to carefully assess existing infrastructure and technology. Infrastructure and technology constituted 30–70% of our project budgets at any one time, depending on the age of the programme, its expansion, etc. Even when an eye care programme is mature and spending a relatively small portion of its budget on infrastructure and equipment, these still represent a large past investment.

As shown in the Box in the next column, you should establish systems for cost-effective procurement, inventory, and monitoring.

We found that staff often required training in the proper storage and handling of assets, as well as in the use of whatever monitoring tools were in place for tracking assets and inventory. Complicated tools and procedures were not well received, so tools should be user-friendly.

## Improving quality

The quality of service delivery and of the patient's experience is dependent on ensuring that the staff are fully aware of the clinical and non-clinical aspects of ‘quality’, and of the role they personally play in improving them.

We cannot overemphasise the impact of improving the quality of clinical and non-clinical services. We found this to be a major driver of service uptake.

In KWECP, we established the concept of ensuring quality at every step of service delivery. We did this by developing a model team in one hospital and by nominating a coordinating consultant who actively trained and supervised ophthalmologists in service.

The programme selected a hospital which had existing infrastructure and human resources, but where output had been low over many years. The staff of this hospital were re-trained in the various clinical and non-clinical skills necessary to improve quality, including human relations. We therefore built a small, but effective, hospital-based eye care team, where responsibilities for administration, outpatient department activities, theatre nursing, etc. were clearly defined. Human resources were also developed in the surrounding communities for primary eye care work and referrals: a community ophthalmic nurse and primary eye care workers were trained, all led and coordinated by the ophthalmologist and his hospital-based team. This hospital- and community-based team was responsible for a population of approximately half a million persons.

The subsequent rapid acceptance and uptake of services and the fast build-up of surgical output from this team served as a positive role model. This model is now being emulated by the rest of the programme and by other programmes supported by Sightsavers in the country.

## Advocacy and management

One of the main roles of the management teams in eye care programmes has been advocacy. Eye care ranks low in priority for health care in most parts of the world and Nigeria is no exception. The programme work in Kaduna, Kwara, and Cross River demonstrated a need for strong advocacy to generate State support and ownership of the eye care plans.

In Sokoto State, the most recent of the eye care plans, major investment was made in advocacy and in advocacy training for staff at the onset of the programme. This approach was highly successful: Sokoto authorities demonstrated much greater ownership and supported programme activities at an earlier stage in programme implementation than was the case for any of the older programmes in other States. The lessons we learnt were:

- consider advocacy as a major activity in your plan
- do not leave advocacy to chance encounters
- advocacy should be carefully planned with set goals and objectives
- invest in advocacy training for key personnel.

## Financial sustainability

Some form of income generation or cost recovery is essential to sustain an eye care programme. The government partners in Nigeria programmes vary in their financial contributions; one State provides regular financial support, while another provides no financial support at all despite extensive advocacy.

It is essential that fees are affordable by the majority of the population. The surgery fees introduced in our programmes have not been a barrier to service delivery and provide resources for maintaining quality of service. Evidence indicates that 80% of patients from the poorest and least developed areas of Sokoto State can afford the fee of about US $12 (half the price of a goat).

Policies should be in place so that no person remains blind on account of his/her inability to pay. In the Nigeria eye care programmes, many are assisted by the local government authorities, some are supported by wealthy members of the community, and others are granted exemption from fees after recommendation by social services.

If free eye camps take place, such as those organised by the State government in Kwara, it is better to organise them in locations far away from the base, as we have noticed that they have a negative impact on paid service delivery in the base hospitals.

**Managing human resources: key points**

1. **Check the available pool and competence level** at the beginning of the human resource development process
2. **Emphasise a team approach**; clarify and develop roles, responsibilities, and reporting channels
3. **Build teams with the correct mix** of professional and support staff
4. **Do not assume existing personnel have the required skills** and knowledge for service delivery
5. **Invest in adequate and continuous training**
6. **Invest in management and advocacy training** for key personnel
7. **A well-motivated team** delivers better results
8. **Recognise individuals** for outstanding performance
9. **Do not single out a single profession** for praise in the case of good performance, or criticism for poor performance (e.g. by saying that low numbers of cataract operations are the fault of the ophthalmologist).

**Managing infrastructure and technology: key points**

1. **Appropriate technology means quality technology at an affordable cost**, so ensure adequate high-quality technology and equipment for service delivery
2. **Do not procure cheap, low-quality instruments or technology**
3. **Institute preventive maintenance**
4. **Establish standards** for technology and consumables
5. **Ensure proper documentation** and tracking of assets
6. **Use tools that are simple and easy to use** for tracking
7. **Do not discard existing systems**, provided that they meet standards
8. **Ensure consumption matches activities** and establish reorder levels.
